# “Have you considered that it could be burnout?”—psychologization and stigmatization of self-reported long COVID or post-COVID-19 vaccination syndrome

**DOI:** 10.1186/s12916-025-04335-0

**Published:** 2025-08-20

**Authors:** Ronja Büchner, Christian Sander, Stephanie Schindler, Martin Walter, Carmen Scheibenbogen, Georg Schomerus

**Affiliations:** 1 Department of Psychiatry and Psychotherapy, Medical Faculty, Leipzig University, Semmelweisstr. 10, Leipzig, 04103 Germany; 2 Department of Psychiatry and Psychotherapy, Medical Center, Leipzig University, Leipzig, Germany; 3 Department of Psychiatry and Psychotherapy, Jena University Hospital, Jena, Germany; 4 Institute of Medical Immunology, Charité - Universitätsmedizin Berlin, corporate member of Freie Universität Berlin and Humboldt Universität zu Berlin, Berlin, Germany

**Keywords:** Long COVID, Post-COVID condition, Stigmatization, Psychologization, Post-COVID-19 vaccination syndrome

## Abstract

**Background:**

People reporting long COVID (LC) or post-COVID-19 vaccination syndrome (PCVS) not only suffer from their symptoms but also from stigmatization. Despite ample account and characterization of stigma experiences so far, its mechanisms and consequences on health outcomes, and particularly the role of “psychologization” remain unclear.

**Methods:**

In a cross-sectional observational study, we examined a large convenience sample of adults who report having LC or PCVS. We translated and adapted the “Long Covid Stigma Scale” to measure stigmatization. We measured generally *perceived* and personally *experienced psychologization* with newly developed scales/items. Outcome measures included disclosure concerns, loss of trust in medicine, life satisfaction, depression, anxiety, self-esteem, and loneliness. We calculated overall prevalences of stigma and psychologization and their correlations with the outcomes. Using mediation analysis with SEM, we tested the hypothesis that psychologization of LC and PCVS syndromes causes harm by increasing stigmatization.

**Results:**

Altogether, *N* = 2053 individuals (68% reporting LC, 32% reporting PCVS) were included in the analyses. The overall prevalences of stigma experiences were high: 83% of those reporting LC and 90% of those reporting PCVS experienced stigma. Prevalences of *perceived psychologization* (LC: 87%, PCVS: 91%) and *experienced psychologization* (LC: 82%, PCVS: 87%) were similarly high. Both stigmatization and psychologization were positively correlated with disclosure concerns, loss of trust in medicine, depression, anxiety, and loneliness as well as negatively correlated with life satisfaction and self-esteem. Mediation analysis indicated that stigmatization mediated a relevant proportion of the relationship between psychologization and negative outcomes.

**Conclusions:**

People reporting LC or PCVS are subject to stigmatization and psychologization. From a patient perspective, psychologization appears to be an important driver of stigmatization and negative outcomes.

**Supplementary Information:**

The online version contains supplementary material available at 10.1186/s12916-025-04335-0.

## Background

People with long COVID (LC) experience a broad range of symptoms affecting the entire body. For example, many struggle with previously unknown states of exhaustion and an increased sensitivity to stimuli of all kinds [1, 2]. Vaccination against COVID-19 reduces the risk of severe courses of acute infections and of developing LC [3–5], but a very small proportion (estimates to date are around 0.003%) of those who have been vaccinated report long-lasting symptoms comparable to LC [6–11]. To date, there is no widely agreed term for the clinical picture of long-lasting symptoms after COVID-19 vaccinations, but following other authors [6, 12], we will use the term “post-COVID-19 vaccination syndrome” (PCVS).

In this article, we want to examine the subjective experiences of people with self-reported LC or PCVS. The focus of this study is thus neither the prevalence nor the pathology of these syndromes. PCVS in particular is still a controversial topic. Public institutions that monitor vaccination safety based on systematically collected data have not found any “safety signal” in their registers and there is no reason to doubt that vaccinations against COVID-19 are generally safe. However, the absence of a safety signal does not equate to the absence of rare individual cases, but rather reflects the current evidence base. Also, it has been argued that there might be other suitable ways to record and evaluate data in pharmacovigilance systems [8]. Clearly, PCVS is an area of ongoing scientific inquiry and further research on prevalence and pathomechanisms is needed to present a differentiated and comprehensive picture.

Besides suffering from the symptoms, many people reporting LC or PCVS also report stigmatizing experiences as well as stigmatization while seeking help. Stigma has been conceptualized as the co-occurrence of labeling, stereotyping, othering, and status loss/discrimination [13]. It is known from other stigmatized conditions like HIV, tuberculosis, epilepsy, or mental illnesses that stigma can impact help-seeking behavior, course and burden of disease, quality of life, and secrecy, as well as feelings of shame, guilt, and loneliness among other negative outcomes [14–17]. The few quantitative studies conducted on stigma and LC found associations of stigma experiences with perceived stress, depressive symptoms, anxiety, quality of life, and disclosure concerns [18, 19]. To our knowledge, there are no studies published on stigma experiences among people reporting PCVS as of today. In this paper, we focus on the stigma experienced by people reporting LC or PCVS, which we measure in terms of enacted, expected, and internalized stigma. Other aspects of stigma, namely stigmatizing attitudes of the public or health care professionals, and structural stigma are beyond the scope of this paper.

A narrative that people reporting LC or PCVS repeatedly encounter is the classification of their symptoms as psychological or at least psychosomatic. A qualitative study by Samper-Pardo et al. contains several statements by individuals reporting LC such as “They tell you that this is all psychological, you are somatizing, go to a mental health clinic” [20]. The symptoms faced by patients are explained both by doctors and people close to them as being a reaction to stress, workload, age, or other challenges. In this study, we define this as “psychologization,” i.e., the *primary causal explanation of a somatic disease with psychological factors*. Our definition also includes *a constant over-emphasis of psychological co-causation*. What we explicitly do not refer to is psychologization in a broader sense as a societal phenomenon, which means the expansion of psychology into all areas of life and the resulting individualization of problems. Even if both interpretations of the term cannot be considered completely independently of each other, in this article, we will focus on the former and mostly neglect the latter.

Psychologization is a common phenomenon for people with myalgic encephalomyelitis/chronic fatigue syndrome (ME/CFS) [21–25]. ME/CFS is a disease that can occur after virus infections like Epstein-Barr, but it can also occur independently of an infection [26–28]. It became more prominent during the pandemic, with around half of LC patients meeting the criteria for ME/CFS at six months, according to a recently published systematic review [29]. To our knowledge, there are no studies on an estimated frequency of ME/CFS in PCVS.

Although many people reporting LC or PCVS have testified about their experiences of psychologization, there is little systematic research on the subject and its associated outcomes. This applies even more to the investigation of the relationship between psychologization and stigmatization. Some qualitative studies suggest a connection between those concepts, but there are no quantitative studies investigating how experiences of psychologization and stigmatization are connected. We assumed that psychologization is one of the important mechanisms behind stigmatization. In other words, we expected that more psychologization leads to more stigmatization and thus psychologization “drives” stigmatization of people reporting LC or PCVS and its harmful effects.

Examining a large sample of people reporting LC or PCVS, we therefore investigated the following research questions:

1. To what extent are people reporting LC or PCVS subjected to psychologization and stigmatization?
2. How are experiences of psychologization and stigmatization related to unfavorable outcomes like disclosure concerns, reduced life satisfaction, and a loss of trust in medicine?
3. Is there evidence for a causal relationship between psychologization and stigmatization of LC and PCVS and individual well-being?

In answer to these questions, we predicted the following findings. First (H1), in line with previous reports about experiences of psychologization and stigmatization reviewed above, we assumed that people reporting LC and PCVS would experience relevant amounts of psychologization and stigmatization of their conditions by other people. Second (H2), we predicted that higher amounts of psychologization and stigmatization of LC and PCVS would be associated with unfavorable outcomes like disclosure concerns, reduced life satisfaction, and a loss of trust in medicine in people experiencing these syndromes. In fact, we expected stigmatization to be one of the primary mechanisms that link psychologization of LC and PCVS to reduced well-being. Accordingly, our third hypothesis (H3) predicted that the harmful effects of psychologization of LC and PCVS would be mediated by stigmatization.

## Methods

### Sample

We conducted an online survey in German addressing a convenience sample of adults aged 18 or above with ongoing symptoms after COVID-19 infection (LC) or COVID-19 vaccination (PCVS). Data collection took place in February 2024. Recruitment was done using a snowball approach by publicizing the access link to the online survey via different patient initiatives throughout Germany. Due to the controversy surrounding LC and PCVS, we avoided recruiting via social media in order to avoid attracting non-affected people or political activists to the survey. The survey was performed in accordance with the Declaration of Helsinki and the rules of good clinical practice. The study was approved by the ethics committee of the Medical Faculty, University of Leipzig (385/23-lk).

### Survey instruments

Besides sociodemographic information, some basic clinical information was collected about LC and PCVS. Participants were asked to indicate the date (month and year) of their initial COVID-19 infection or their vaccination as well as the start of LC/PCVS symptoms (month and year), and whether a formal diagnosis of LC/PCVS had been made by a doctor (“expert diagnosis”). Thus, information on the diseases and also on the confirmed diagnoses was based exclusively on self-reporting and was not independently confirmed. To determine the severity of the disease (at two different times: at the worst stage and at the time of the survey), we used the Post COVID Syndrome Score [30], and applied it to PCVS. Based on reported symptom clusters, the Post COVID Syndrome Score divides the severity of the disease into three categories. Each of the twelve symptom clusters is assigned a value by which it is multiplied (e.g., fatigue × 7, gastrointestinal complaints × 5, skin complaints × 2). A composite score between 0 and 59 can be achieved, whereby up to 10.75 is considered “mild,” between 10.75 and 26.25 “moderate,” and over 26.25 “severe.”

To measure *stigmatization*, we translated and adapted the “Long Covid Stigma Scale” [18], which was developed in the UK with the involvement of people with lived experience of LC. The scale addresses three categories of stigma experiences (enacted, internalized, and anticipated stigma), each with four to five items. We translated the items into German and, with the help of a native speaker, did a back-translation to ensure that the meaning of the item content was retained (see Additional File 1: Table S1 for the translated items). We did some adaptations to the scale format (see Additional File 2, paragraph 1 for details). For the subsample reporting PCVS, we used a modified wording of the items, replacing “Long COVID” with “Post Vac”, which is a common German term for PCVS. However, in line with other authors [6], we do not recommend using the term in scientific discourse. The internal consistency of the adapted LCSS was high, with Cronbach’s alpha α = 0.88. It is possible to use the three subscales separately or the sum score referring to “stigma experiences” in general.

The authors of the LCSS defined two stigma prevalence scores [18]. First, an estimated prevalence which includes all respondents who answer at least “sometimes” to at least one question within the overall stigma scale and each sub-scale. The second score is more conservative and only includes the respondents who choose the two upper response categories “often” and “very often” in one or more individual questions within the overall stigma scale and each sub-scale. For our sample, we report the conservative prevalence score and, in brackets, the more liberal prevalence score.

To measure *perceived psychologization*, we created six items that addressed aspects of the respondent’s perception of psychologization in the context of their disease. The items (e.g., “I get the impression that many people see LC/PCVS as a purely mental illness”) could be answered on the same 5-point agreement scale (0 = “strongly disagree”; 1 = “somewhat disagree”; 2 = “neither agree nor disagree”; 3 = “somewhat agree”; 4 = “strongly agree”) as the adapted LCSS items. As two items turned out to be unsuitable based on statistical criteria (see Additional File 1: Table S2 for details), we removed them and calculated the total score based on the four remaining items instead. The internal consistency of the final four-item scale was high; Cronbach’s alpha was *α* = 0.86. The prevalence score for perceived psychologization was calculated in accordance with a principle similar to the LCSS’s conservative stigmatization score. It includes the respondents who chose the two upper response categories “somewhat agree” and “strongly agree” in one or more individual questions within the overall scale.

We also asked for *experienced psychologization*: “Have you ever experienced your LC/PCVS symptoms primarily being attributed to a mental illness? (e.g., “burnout”, depression)”. Answers could be given on a 5-point rating scale ranging from 1 = “never” (2 = “rarely”; 3 = “sometimes”; 4 = “often”) to 5 = “very often.” Furthermore, participants should indicate from whom they had experienced psychologization (e.g., medical-therapeutic professionals, family, friends; multiple answers were possible) and how strongly they felt emotionally burdened. The level of emotional burden could also be rated on a 5-point rating scale ranging from 1 = “not at all” to 5 = “very strong”; we considered ratings 4 and 5 as emotionally burdened.

To control for potential effects on psychologization and stigmatization, survey participants could indicate whether they had any personal *history of mental illness* (over their lifespan).

*Loss of trust in medicine.* We further asked the respondents to rate a potential change in their trust in medicine on a 9-point scale anchored by the labels 1 “strongly decreased,” 5 “unchanged,” and 9 “strongly increased.” Given that the majority of our respondents indicated a decrease in trust (marked right-tailed distribution of the responses), we inverted the item scale prior to statistical analysis so that high values represent a loss of trust in medicine.

We screened for *depressive mood and anxiety* using the PHQ-4 [31]. Since using the PHQ-9 and comparable instruments is a source of problems due to the symptom overlap with LC/PCVS, we preferred the short PHQ-4. In the analysis, we analyzed them separately as PHQ-2 and GAD-2, each covering two core symptoms of depression and anxiety. Additionally, participants could indicate their overall level of *life satisfaction*, which was measured by a single-item, ranging from 0 to 10 [32].

*Self-esteem* was assessed with the Brief Rosenberg Self-Esteem Scale [33], which consists of the five most informative items of Rosenberg’s Self-Esteem Scale, and *Loneliness* with the Three-Item Loneliness Scale [34]. Scores were calculated for the questionnaires in accordance with the respective evaluation instructions.

Following the authors of the LCSS [18], the assessment of *disclosure concerns* was addressed with two items on caution and regrets of sharing the medical condition with others. For further calculations, the two items, ranging from 1 to 5, were summarized.

### Data analysis

#### Analysis sample

Between January, the 31st, and February, the 14th, 2024, the survey link was used 3404 times. A total of *N* = 2,689 participants gave their consent and were forwarded to the survey. However, *N* = 2537 participants proceeded to answer the first question. Some participants had to be excluded for not finishing the questionnaire (*n* = 422), not having persistent symptoms (*n* = 40), not being of legal age (*n* = 7), not passing an attention check (*n* = 3), or other reasons (*n* = 12); see Additional File 2, paragraph 2 for details.

#### Statistical analysis

We used R (version 4.4.1) for all statistical analyses except for the initial data curation and characterization of the six items on perceived psychologization (IBM SPSS Statistics, Version 29.0). As all study measures were either categorical or deviated from a normal distribution (visual inspection of the density distribution plus Shapiro-Francia normality test), we report nonparametric descriptive statistics (proportions or median with interquartile range – IQR, respectively). Analogously, group comparisons employed chi-square tests for categorical data and Wilcoxon’s signed ranks test (Mann–Whitney *U*-test, respectively) for continuous variables.

**Fig. 1:**
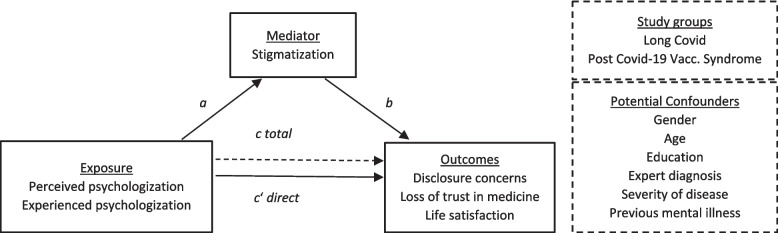
Proposed causal mechanisms of psychologization of the long COVID syndrome and post-COVID-19 vaccination syndrome. Structural equation model specification for the proposed relationship between psychologization and stigmatization of long COVID or post-COVID-19 vaccination syndromes, and negative outcomes. Arrows represent path coefficients—dashed before inclusion of the mediator (i.e., bivariate relationship) and labelled in accordance with Table 4

Details of our analyses are given in Additional File 2, paragraph 3 [35–37]. For hypothesis H1, we report proportions of agreement for each psychologization item (perceived or experienced) and prevalence rates of stigmatization in accordance with the dichotomization instructions of the original authors of the LCSS [18]. To answer hypothesis H2, we estimated the association of psychologization and stigmatization with negative outcomes (disclosure concerns, reduced life satisfaction, and a loss of trust in medicine) using Spearman’s correlation coefficient Rho for ordinal data. We tested our third hypothesis H3, predicting that the actual cause of these negative effects is psychologization, which increases stigmatization and, through that, reduces well-being, via a mediation analysis using structural equation modeling (SEM) provided by the lavaan package; version 0.6–18 for R [38]. The mediation model comprised psychologization as the exposure variable, stigmatization as the mediator, and the three outcomes, as well as relevant covariates illustrated in Fig. 1 and detailed in Additional File 2. We report standardized coefficients and *p*-values corrected for multiple testing using Bonferroni’s procedure.

## Results

### Sample characteristics

Our final analysis sample included* N* = 2,053 respondents, of whom *N* = 1,398 (68%) stated that they suffered from persistent symptoms resulting from a COVID-19 infection (LC subsample), while N = 655 (32%) reported that their symptoms had developed after a COVID-19 vaccination (PCVS subsample). Overall, 83% described themselves as female, 16% as male, and eight respondents indicated a non-binary gender. There were significantly more women in the LC group than in the PCVS group (+ 9%, *χ*^2^ (2, *N* = 2053) = 27.2, *p* < 0.001). The median age in the total sample was 43 years (IQR: 34–52 years) and comparable across both study groups (*W* = 345,846, *p* = 0.08, Wilcoxon rank sum test). Our respondents were highly educated, with 70% reporting degrees equivalent to American high schools or universities (more than 10 years of education) and 24% reporting 10 years of education. A comparison of the two study groups indicated a slightly higher education in the LC group at the threshold level of statistical significance (*χ*^2^ (2, *N* = 2031) = 6.69, *p* = 0.035). The regional origin of our sample was well distributed across all federal states of Germany, with additionally 5.3% of the LC group and 4.3% of the PCVS group participating in countries neighboring Germany.

Overall, 79% of our participants reported that a medical doctor established the diagnosis of LC or PCVS, 25% more in the LC group than in the PCVS group (*χ*^2^ (1, *N* = 2053) = 169.0, *p* < 0.001). The number of symptoms in the total sample was high, with a median Syndrome Score of 41 (IQR = 33–49) out of a possible 59 points. Although this value was only 3 points higher in the LC than in the PCVS group, a Wilcoxon rank sum test indicated this difference to be highly statistically significant (LC: median = 42, IQR = 33–50; PCVS: median = 39, IQR = 31–49, *W* = 403,419, *p* < 0.001). In the total sample, the reported causative incidents (i.e., infection or vaccination) had occurred a median of 25 months (IQR = 20–33) prior to our study. However, in the LC subsample, the COVID-19 infection was reported to have occurred a median of 23 months ago (IQR = 17–28) whereas in the PCVS group, the vaccination was reported to have been received a median of 30 months ago (IQR = 26–33, *W* = 661,002, *p* < 0.001, Wilcoxon rank sum test). For detailed sample characteristics, please refer to Table 1.

**Table 1:** Sample characteristics

	Total sample (*N* = 2053)	LC subsample (*N* = 1398)	PCVS subsample (*N *= 655)
Gender identity—*n* (%)			
Male	335 (16.0)	187 (13.4)	148 (22.6)
Female	1710 (83.0)	1203 (86.1)	507 (77.4)
Other	8 (0.4)	8 (0.6)	0 (0.0)
Age—median (IQR)	43 (34–52)	44 (34–53)	42 (33–52)
Education—*n* (%)			
Short (< 10 years)	94 (4.6)	55 (3.9)	39 (6.0)
Medium (= 10 years)	501 (24.4)	330 (23.6)	171 (26.1)
Long (> 10 years)	1436 (69.9)	1000 (71.5)	436 (66.6)
unspecified	22 (1.1)	14 (0.9)	9 (1.4)
Months since COVID-19 infection/vaccination—median (IQR)	25 (20–33)	23 (17–28)	31 (26–33)
Expert diagnosis—*n* (%)			
Yes	1630 (79.4)	1221 (87.3)	409 (62.4)
No	346 (16.9)	134 (9.6)	212 (32.4)
Do not know	77 (3.8)	43 (3.1)	34 (5.2)
Disease severity (worst phase)—*n* (%)			
Mild	20 (1.0)	12 (0.9)	8 (1.2)
Moderate	247 (12.0)	142 (10.2)	105 (16.0)
Severe	1786 (87.0)	1244 (89.0)	542 (82.7)
Disease severity (currently)—*n *(%)			
Mild	116 (5.7)	64 (4.6)	52 (7.9)
Moderate	547 (26.6)	362 (25.9)	185 (28.2)
Severe	1390 (67.7)	972 (69.5)	418 (63.8)
Symptom severity (worst phase)—median (IQR)	41 (33–49)	42 (33–50)	39 (31–49)
Symptom severity (currently)—median (IQR)	33 (24–42)	33 (24–42)	33 (22–43)
Previous mental illness—*n* (%)			
Yes	811 (39.5)	609 (43.6)	202 (30.8)
No	1177 (57.3)	749 (53.6)	428 (65.3)
Do not know	23 (1.1)	15 (1.1)	8 (1.2)
Do not want to say	42(2.1)	25 (1.8)	17 (2.6)

Sociodemographic and clinical characteristics of the total sample and subsamples

*IQR *interquartile range, *LC* long COVID, *n.a.* not available, *PCVS *post-COVID-19 vaccination syndrome

### Extent of psychologization and stigmatization (H1)

In line with our first hypothesis (H1), the majority of our respondents indicated psychologization and stigmatization of their reported LC and PCVS diseases by other people.

#### Perceived psychologization

**Fig. 2:**
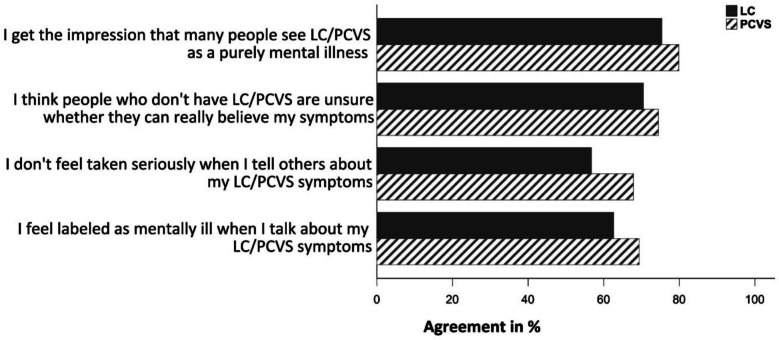
Frequency of perceived psychologization among people reporting long COVID or post-COVID-19 vaccination syndrome. Proportion of affirmative responses (“somewhat agree” and “strongly agree”) to newly designed items about perceived psychologization among people reporting long COVID syndrome (LC; *N* = 1398) and post-COVID-19 vaccination syndrome (PCVS; *N* = 655)

The prevalence of perceived psychologization was 88% in the full sample, 87% for participants reporting LC and 91% for those reporting PCVS. General agreement, i.e., ratings “somewhat agree” and “strongly agree” with our selection of four newly designed items ranged from 60% (“I don’t feel taken seriously when I tell others about my LC/PCVS symptoms”) to 77% (“I get the impression that many people see LC/PCVS as a purely mental illness”) in the full sample. In the LC subgroup, agreement with these items was descriptively slightly lower (range: 57–76%) than in the PCVS subgroup (68–80%, see Fig. 2). The composite (average) score of all four items had a median of 3.0 in the full sample (IQR: 2.3–3.8) as well as in the LC subgroup (IQR: 2.3–3.5), and a median of 3.3 (IQR: 2.5–3.8) in the PCVS subgroup (please refer to Table 2).

**Table 2:** Descriptive statistics of the study measures

	Total sample (*N* = 2053)	LC subsample (*N* = 1398)	PCVS subsample (*N* = 655)
Perceived psychologization*	3.0 (2.3–3.8)	3.0 (2.3–3.5)	3.3 (2.5–3.8)
Experienced psychologization	4 (3–5)	4 (3–4)	4 (3–5)
LCSS-total stigma*	2.0 (1.5–2.6)	2.0 (1.5–2.5)	2.2 (1.5–2.8)
LCSS enacted stigma*	1.6 (1.0–2.4)	1.6 (0.8–2.2)	2.0 (1.2–2.8)
LCSS internalized stigma*	2.0 (1.0–2.8)	2.0 (1.3–2.8)	1.8 (1.0–2.8)
LCSS anticipated stigma*	2.5 (2.0–3.3)	2.5 (2.0–3.3)	2.8 (2.0–3.3)
Disclosure concerns*	3.0 (2.0–4.0)	3.0 (2.0–4.0)	3.0 (2.0–4.0)
Loss of trust in medicine	8 (6–9)	7 (6–8)	9 (8–9)
Life-satisfaction (L1)	3 (2–5)	3 (2–5)	3 (1–6)
Depression (PHQ-2)	2 (1–4)	2 (1–3)	2 (1–4)
Anxiety (GAD-2)	2 (1–3)	2 (1–3)	2 (1–4)
Self-esteem (BRSES)*	5.6 (4.4–6.4)	5.6 (4.4–6.4)	5.8 (4.6–6.6)
Loneliness (UCLA-3)	7 (5–9)	7 (6–9)	6 (5–9)

Distributional characteristics (median and interquartile range) of the study measures in the study sample and its subgroups

*BRSES* Brief Rosenberg Self-esteem Scale, *GAD-2*Generalized Anxiety Disorder (2 items), *LC* long COVID, *LCSS* Long Covid Stigma Scale, *L1 *Single Item Life-Satisfaction, *PCVS* post-COVID-19 vaccination syndrome, *PHQ-2* Patient Health Questionnaire (2 items), *UCLA-3* Loneliness Scale

*Mean score

#### Experienced psychologization

With respect to our single item on experienced psychologization, 84% of all study participants, 82% of the participants in the LC group, and 87% in the PCVS group reported having experienced psychologization at least sometimes. The median response to this item was 4 “often” in the total sample (IQR: 3–5), as well as in the LC group (IQR: 3–4) and in the PCVS group (IQR: 3–5). The correlation between the composite score of perceived psychologization and the single item on experienced psychologization was ϱ = 0.59 in the full sample, ϱ = 0.61 in the LC group, and ϱ = 0.53 in the PCVS group, representing strong associations. A large majority (total: 92%) of those respondents who reported experiences of psychologization on at least some occasions attributed these to doctors and medical-therapeutic professionals (LC: 90% and PCVS: 96%). Experiences of psychologization were also oftentimes caused by friends (total: 47%, LC: 49%, and PCVS: 43%), public authorities (total: 41%, LC: 38%, and PCVS: 48%), and family members (total: 41%, LC: 42%, and PCVS: 39%). In contrast, the perceived emotional burden of experienced psychologization (ratings 4 and 5 on the 5-point rating scale) was very similar across these different agencies: medical staff: 92% (LC: 91% and PCVS: 93%), friends: 82% (LC 81% and PCVS: 84%), public authorities: 85% (LC: 85% and PCVS: 86%), and family members: 85% (LC: 84% and PCVS: 85%).

#### Stigmatization

The overall prevalence of stigmatization reported in the total sample was 89% (liberal estimate: 96%). Looking separately at the three stigmatization facets, the prevalence rates were 50% (84%, respectively) for enacted stigma, 67% (87%) for internalized stigma, and 85% (96%) for anticipated stigma. For people reporting LC, overall stigma prevalence was 83% (96%). Regarding the facets, the prevalence rates were 44% (82%) for enacted stigma, 68% (88%) for internalized stigma, and 83% (96%) for anticipated stigma. For respondents reporting PVCS, the prevalence rates usually exceeded those of the LC group, with prevalence rates of 90% (96%) for the full LCSS, 64% (90%) for enacted stigma, 63% (82%) for internalized stigma, and 88% (98%) for anticipated stigma. The composite (average) LCSS score had a median of 2.2 (IQR: 1.5–2.6) in the total sample as well as in the LC group (IQR: 1.5–2.5), and a median of 2.2 (IQR: 1.5–2.8) in the PCVS group. For further descriptive statistics on the LCSS subscale scores, please refer to Table 2.

### Relation of psychologization and stigmatization with outcomes (H2)

In line with our second hypothesis, higher amounts of both psychologization as well as stigmatization were correlated with unfavorable outcomes. Stigmatization was positively correlated with disclosure concerns, with Spearman’s ϱ = 0.49, *p* < 0.001 (full sample); correlation was on the immediate threshold between medium and strong [39]. Furthermore, stigma was positively correlated with loss of trust in medicine (full sample: ϱ = 0.31, *p* < 0.001) and loneliness (ϱ = 0.38, *p* < 0.001), as well as depression (ϱ = 0.28, *p* < 0.001) and anxiety (ϱ = 0.28, *p* < 0.001). Small to moderate negative correlations were found for life satisfaction (ϱ =  − 0.24, *p* < 0.001) and self-esteem (ϱ =  − 0.30, *p* < 0.001).

Similar patterns emerged for both perceived and experienced psychologization: positive correlations could be observed with disclosure concerns, loss of trust in medicine, depression, anxiety, and loneliness, while negative correlations were found for life satisfaction and self-esteem. Effect sizes ranged from small for experienced psychologization and self-esteem (ϱ =  − 0.11, *p* < 0.001) to medium with a tendency to strong for perceived psychologization and disclosure concerns (ϱ = 0.46, *p* < 0.001). Please see Table 3 for a comprehensive description of all bivariate correlation coefficients of psychologization and stigmatization and the outcome measures both in the full sample and subsamples.

**Table 3:** Associations between psychologization, stigmatization, and well-being in people reporting long COVID or post-COVID-19 vaccination syndrome

		Perceived psychologization	Experienced psychologization	LCSS total stigma	LCSS enacted stigma	LCSS internalized stigma	LCSS anticipated stigma
Total sample (*N* = 2053)	Disclosure concerns	0.46***	0.31***	0.49***	0.39***	0.36***	0.48***
	Loss of trust in medicine	0.29***	0.33**	0.31***	0.36	0.12***	0.28***
	Life-satisfaction (L1)	− 0.16***	− 0.15***	− 0.24***	− 0.18***	− 0.24***	− 0.17***
	Depression (PHQ2)	0.27***	0.17***	0.28***	0.14***	0.33***	0.22***
	Anxiety (GAD2)	0.27***	0.18***	0.28***	0.13***	0.35***	0.22***
	Self-esteem (BRSES)	− 0.21***	− 0.11***	− 0.30***	− 0.08*	− 0.47***	− 0.20***
	Loneliness (UCLA3)	0.19***	0.16***	0.38***	0.32***	0.33***	0.26***
LC subsample (*N* = 1398)	Disclosure concerns	0.50***	0.34***	0.52***	0.43***	0.37***	0.49***
	Loss of trust in medicine	0.31***	0.34***	0.33***	0.34***	0.16***	0.32***
	Life-satisfaction (L1)	− 0.16***	− 0.14***	− 0.24***	− 0.18***	− 0.25***	− 0.17***
	Depression (PHQ2)	0.26***	0.16***	0.28***	0.13***	0.33***	0.22***
	Anxiety (GAD2)	0.26***	0.17***	0.29***	0.12***	0.36***	0.23***
	Self-esteem (BRSES)	− 0.22***	− 0.10*	− 0.30***	− 0.10**	− 0.45***	− 0.20***
	Loneliness (UCLA3)	0.18***	0.14***	0.37***	0.36***	0.30***	0.23***
PCVS subsample (*N* = 655)	Disclosure concerns	0.38***	0.23***	0.44***	0.32***	0.34***	0.46***
	Loss of trust in medicine	0.21***	0.22***	0.26***	0.28***	0.14*	0.20***
	Life-satisfaction (L1)	− 0.16**	− 0.16***	− 0.23***	− 0.16**	− 0.22***	− 0.16**
	Depression (PHQ2)	0.26***	0.16**	0.26***	0.12	0.33***	0.20***
	Anxiety (GAD2)	0.28***	0.19***	0.27***	0.12	0.36***	0.20***
	Self-esteem (BRSES)	− 0.22***	− 0.17***	− 0.31***	− 0.08	− 0.49***	− 0.19***
	Loneliness (UCLA3)	0.27***	0.23***	0.44***	0.35***	0.37***	0.35***

Bivariate correlation coefficients (Spearman’s ϱ) with asterisks representing *p*-values corrected for multiple testing (42 tests, Bonferroni’s procedure)

*BRSES* Brief Rosenberg Self-esteem Scale, *GAD-2* Generalized Anxiety Disorder (2items), *LC* long COVID, *LCSS* Long Covid Stigma Scale, *L1* Single Item Life-Satisfaction, *PCVS* post-COVID-19 vaccination syndrome, *PHQ-2 *Patient Health Questionnaire (2 items), *UCLA-3* Loneliness Scale

*** *p* < .001, ** *p* < .01, * p < .05

### Evidence for a causal relationship between psychologization, stigmatization, and reduced well-being (H3)

**Fig. 3:**
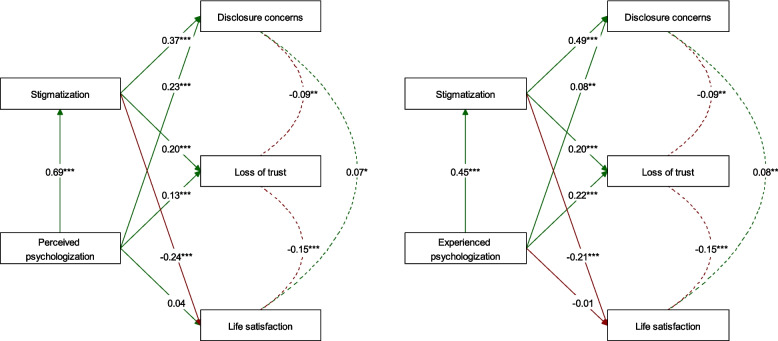
Pathways between perceived or experienced psychologization, stigmatization, and well-being in people reporting long COVID or post-COVID-19 vaccination syndrome. Results of the mediation analysis in the full sample (*N* = 2053) of participants reporting long COVID or post-COVID-19 vaccination syndrome, controlled for gender, age, education, expert diagnosis of the syndrome, syndrome severity, and previous mental illness. Arrows represent standardized regression coefficients and dashed curves indicate covariances between the outcomes. Asterisks represent *p*-values corrected for separate testing of two exposures and three outcomes (Bonferroni’s procedure). *** *p* < 0.001, ** *p* < 0.01, * p < 0.05

The results of the mediation analysis are summarized in Table 4 and illustrated in Fig. 3. First, we examined the total effect in the full sample, which is analogous to the bivariate relationships reported above. The associations between perceived psychologization and disclosure concerns, loss of trust in medicine, and reduced life satisfaction were of small to medium effect size (*B* = 0.48, 0.27, and − 0.12, all *p* < 0.001), confirming the nonparametric estimates.

**Table 4:** Stigmatization as mediator of the detrimental effects of psychologization of the long COVID or post-COVID-19 vaccination syndrome

Sample	Outcome	Exposure-mediator (*a*)	Mediator-outcome (*b*)	Direct effect (*c*’)	Mediation (*a* * *b*)	Total (*c*′ + *a* * *b*)	*R*^2^
Exposure perceived psychologization							
Total sample (*N* = 2053)	Disclosure concerns	0.69***	0.37***	0.23***	0.25***	0.48***	0.30
	Loss of trust in medicine		0.20***	0.13***	0.14***	0.27***	0.14
	Life satisfaction		− 0.24***	0.04	− 0.17***	− 0.12***	0.08
LC subsample (*N* = 1398)	Disclosure concerns	0.71***	0.35***	0.27***	0.25***	0.51***	0.33
	Loss of trust in medicine		0.21***	0.15**	0.15***	0.30***	0.15
	Life satisfaction		− 0.25***	0.04	− 0.17***	− 0.13***	0.08
PCVS subsample (*N* = 655)	Disclosure concerns	0.64***	0.41***	0.14*	0.26***	0.40***	0.27
	Loss of trust in medicine		0.14*	0.11	0.09*	0.20***	0.12
	Life satisfaction		− 0.23***	0.06	− 0.15***	− 0.09	0.09
Exposure experienced psychologization							
Total sample (*N* = 2053)	Disclosure concerns	0.45***	0.49***	0.08**	0.22***	0.30***	0.28
	Loss of trust in medicine		0.20***	0.22***	0.09***	0.31***	0.17
	Life satisfaction		− 0.21***	− 0.01	− 0.09***	− 0.10***	0.08
LC subsample (*N* = 1398)	Disclosure concerns	0.49***	0.50***	0.09**	0.24***	0.33***	0.30
	Loss of trust in medicine		0.22***	0.21***	0.11***	0.32***	0.17
	Life satisfaction		− 0.23***	0.01	− 0.11***	− 0.10**	0.08
PCVS subsample (*N* = 655)	Disclosure concerns	0.34***	0.48***	0.07	0.16***	0.23***	0.27
	Loss of trust in medicine		0.17***	0.16**	0.06***	0.21***	0.13
	Life satisfaction		− 0.18***	− 0.04	− 0.06**	− 0.10	0.09

Results of the mediation analysis of negative effects of psychologization of long COVID or post-COVID-19 vaccination syndrome. We report standardized regression coefficients with asterisks representing *p*-values corrected for multiple testing (six tests per sample; Bonferroni’s procedure)

*** *p* < .001, ** *p* < .01, * p < .05

We then inspected the two pathways additively responsible for these relationships in the full sample. On one side, perceived psychologization explained almost half of the variance (48%) in stigmatization (*B* = 0.69, *p* < 0.001, medium effect size), and the latter explained between 4 and 14% of the variance in the three outcomes (*B* = 0.37, 0.20, and − 0.24, small to medium effect sizes, all *p* < 0.001). Linked together, both effects explained more than half of the total relationship between perceived psychologization and negative outcomes (proportion total effect mediated > 50% each), suggesting that stigmatization is an important mechanism by which perceived psychologization of LC and PCVS reduces well-being in people reporting these syndromes.

On the other side, the concurrent direct effects of perceived psychologization on the outcomes disclosure concerns and loss of trust in medicine remained robustly significant (*B* = 0.23 and 0.13, respectively; both *p* < 0.001), suggesting additional, yet unknown mechanisms by which perceived psychologization affects these two outcomes (partial mediation). In contrast, for the outcome life satisfaction, the direct effect of perceived psychologization was reduced to non-significance (*B* = 0.04, *p* > 0.05), which, considering the large sample size and hence adequate power, implies a full mediation of the effect of perceived psychologization on life satisfaction by stigmatization.

These patterns observed in the full sample were perfectly replicated in the LC subgroup and, occasionally with descriptively smaller effects, in the PCVS subgroup. Interestingly, the mediation model also was well replicated with experienced psychologization as alternative exposure, despite the fact that the responses to this single item predicted stigmatization less effectively (20% explained variance in the full sample, *B* = 0.45, *p* < 0.001, small effect size). Finally, the mediation model also showed adequate robustness in the sensitivity analysis under exclusion of the two potentially semantically confounded stigmatization items (please see Additional File 1: Table S3).

## Discussion

Our study found that people reporting LC or PCVS face a large amount of stigmatization and psychologization of their symptoms. More disclosure concerns, a loss of trust in medicine, higher rates of depression, anxiety, and loneliness, as well as lower self-esteem and life satisfaction were all related to both psychologization and stigmatization. Furthermore, we observed a clear link between psychologization and stigmatization in the sense that psychologization causes harm to the well-being of patients via increasing stigma.

Before discussing these results in detail, it is worth considering the strengths and limitations of our study. First of all, an important limitation is that our data on diagnoses is based on self-report and could not be verified. We cannot exclude the possibility that memories of triggering events of the symptoms are subject to bias. However, we observed no indications suggesting deliberate misrepresentation. As this study did not collect data based on confirmed diagnosis, we cannot substantiate our judgment further. In this study, we focus on the subjective experience of respondents, examining the correlates of experiences of stigma and devaluation, which have been widely reported by both groups. For reliable diagnosis of LC or PCVS, other ways of recruiting would have been more suitable. In fact, we assume that the percentage of individuals reporting PCVS is significantly overrepresented in our study. One possible reason for the dedicated participation of this group in the study could be that there has not been any research on PCVS and stigma so far, even in comparison to LC. Either way, our data cannot be interpreted as evidence of incidence or causality.

As a second limitation, we examine experiences and perceptions of people reporting LC or PCVS; thus, we do not know how the stigmatizing attitudes and practices people experience are distributed both in the general population and among health care professionals. A clear strength of the study is the large number of participants spread across all German federal states. We can therefore assume that the data is fundamentally meaningful, even if the sample was convenient and not representative, and could therefore be subjected to sampling bias. This is also supported by the fact that gender distribution was similar to other studies on LC, and sample characteristics were generally similar to the sample used in the study by Pantelic et al. in the UK, which was recruited via special LC clinics [18]. Still, prevalences of stigma experiences and experiences of psychologization are not representative and could be lower in other groups of people reporting LC or PCVS not included in this study. Therefore, we could not answer research question 1 conclusively. Regarding the causal relationship between psychologization, stigmatization, and outcomes, the limits of cross-sectional studies also apply to our study. We cannot exclude reverse causality, but the similar results regarding both experienced and perceived psychologization seem to validate our findings. Nor can we exclude confounding of our models by an unmeasured variable. However, we controlled our models for a wide range of potential confounders, including illness severity, and found relations to be robust and meaningful.

Overall, we have observed significant rates of stigmatization, as well as both perceived and experienced psychologization. Our finding that experienced psychologization is clearly led by psychologization from doctors in terms of frequency, as well as emotional impact, is in line with the existing qualitative research on stigma of LC [20, 40]. The observed overall high levels of emotional burden accompanying experiences of psychologization show that psychologization represents an additional burden for people reporting LC or PCVS.

We assumed a clear connection between stigmatization and psychologization and claimed psychologization as one of the important mechanisms behind stigmatization. From the perspective of people reporting LC or PCVS, our data clearly support this hypothesis. The calculated SEM showed that relevant parts (up to 50%) of the psychologization effects on three outcomes considered in more detail (disclosure concerns, trust in medicine, and life satisfaction) were mediated by stigmatization. In other words, more psychologization led to more stigmatization and thus perceived and experienced psychologization “drove” the experience of stigma and its harmful effects. We consider this a highly relevant finding as it questions the widely held assumption that psychologization does not do much harm in the context of LC and PCVS.

On the contrary, our data indicates a clearly harmful nature of psychologization both on a personal and on a structural level. On a personal level, we found many unfavorable outcomes linked to psychologization both directly and indirectly via stigmatization. On a structural level, the psychologization as a driver of stigmatization might play a role in preventing systematic, biomedical research. To some extent, this might also explain the shortage of comprehensive, quality-assured medical care for people reporting LC or PCVS: As long as a strong psychological (co-)causation is assumed (= psychologization), there is no urgent need for biomedical research (= structural stigmatization). A large qualitative evaluation of a German health insurance portal, which records the medical and social care experiences of LC patients, points out the same: “The psychologization of post-viral symptoms is described as stigmatizing and considered the main cause of the precarious care situation of those affected by long COVID” [41]. The harmful effects of psychologization have been studied in other patient populations. A qualitative study on people with rheumatism found being misdiagnosed with a psychosomatic or psychiatric disorder was associated with a damaged self-worth and lower levels of satisfaction with medical care [42]. König et al. [43] studied the occurrence of suicidal thoughts in ME/CFS patients in Switzerland and identified being told the disease is psychosomatic as the factor contributing most to suicidal thoughts [43]. Another important factor was stigmatization as a result of ME/CFS. Regarding suicidal thoughts, both psychologization and stigmatization played a bigger role than, e.g., social isolation or financial stress. Importantly, the suicidal thoughts occurred completely independent of psychiatric comorbidities like depression. Again, this shows the relevance and the worrying nature of our findings as it is reasonable to assume that the results by König et al. may be directly transferable to people reporting LC and PCVS, not least because around half of people with LC meet the ME/CFS criteria after 6 months [29].

Psychologization of post-viral diseases leads to patients being left to their own devices or referred to a psychiatrist or psychotherapist. The implications of treatment following the view through a psychological lens could be harmful and have serious consequences for those affected, especially in the presence of post-exertional malaise (PEM), the core symptom of ME/CFS [22, 23, 44]. The risk of deterioration due to incorrect treatment with activating methods such as graded exercise therapy (GET) or standard cognitive behavioral therapy (CBT) is real and must be taken into account when psychotherapy is being considered [44]. It must be clear that psychotherapy has a supportive and not a curative function for people with post-viral diseases like ME/CFS and LC or PCVS. If the role of psychotherapy is considered in this sense, professional psychotherapeutic support can be helpful for some patients in coping with their disease, comparable with supportive therapy during cancer treatment.

### Implications for clinical practice

Knowledge on pathomechanisms of the clinical pictures summarized under the umbrella term of LC is growing rapidly, and—more slowly—also the insights on PCVS. A mental illness genesis is clearly not tenable. It is now an important task for politicians and the healthcare system to bring this growing knowledge into primary care such as GP’s offices. Practitioners need to be conscious of the detrimental experiences most patients have had within the healthcare system and need to avoid adding to the burden of stigma. Rejection of perceived psychologization by patients should not prematurely be framed as “resistance” or “denial.” Instead, carefully listening to the patients and professional humility both regarding the patients’ experiences and the rapidly evolving knowledge about LC and PCVS is warranted. Lack of knowledge about pathomechanisms should not be an excuse for a “deus ex machina” psychologization of the condition, but rather a call for continuing education and research. Psychiatrists, psychotherapists, and neurologists could also play an important role in differential diagnostics, provided that they have an in-depth knowledge on post-viral diseases.

### Future directions

Our research covers the perspective of people reporting LC or PCVS. What is missing is the perspective of care providers such as doctors. There are many open questions on the perceived exerted extent and possible reasons for stigmatization and psychologization in that regard. Future research should address these questions, also to find approaches for change to minimize the additional burden caused by stigma and psychologization. The same applies to the perspective of the general population on LC and PSCS. There is still a long way to go in understanding the complex mechanisms and interactions of stigma and psychologization surrounding LC, PCVS, and overlapping diseases like ME/CFS. Especially for those affected by the latter, it would be essential to benefit from the current focus of attention after decades of neglect.

## Conclusions

To our knowledge, this is the first quantitative study conducted on stigmatization and psychologization and their relation to each other in people reporting LC and PCVS. For both groups, we observed high levels of stigmatization and both perceived and experienced psychologization. Higher ratings of stigmatization and psychologization correlated with many unfavorable outcomes like a loss of trust in medicine, a higher number of disclosure concerns, and lower life satisfaction. Relevant parts of the relationship between psychologization and outcomes were mediated by stigmatization, so from a patient perspective, psychologization appears to be an important driver of stigmatization and its negative outcomes.

## Supplementary Information

Additional File 1: Tables S1–S3. TblS1 – [German translation LCSS]. TblS2 – [Characteristics of the initial six items of perceived psychologization]. TblS3 – [Analysis of sensitivity: semantic confounding].

Additional File 2: Additional information on Methods.

Additional File 3: Original survey.

## Data Availability

The data that support the findings of this study are available from the corresponding author, upon reasonable request.

## Abbreviations

LC: Long COVID
PCVS: Post-COVID-19 vaccination syndrome
ME/CFS: Myalgic encephalomyelitis/chronic fatigue syndrome
LCSS: Long Covid Stigma Scale
