# Supplementary material for: “Have you considered that it could be burnout?”—psychologization and stigmatization of self-reported long COVID or post-COVID-19 vaccination syndrome
Source: BMC Med. 2025 Aug 20;23:488. doi: 10.1186/s12916-025-04335-0 (PMC12366089; doi:10.1186/s12916-025-04335-0)
Supplement: Supplementary file 1 — Additional File 1: Tables S1–S3. TblS1 – [German translation LCSS]. TblS2 – [Characteristics of the initial six items of perceived psychologization]. TblS3 – [Analysis of sensitivity: semantic confounding]. [file 12916_2025_4335_MOESM1_ESM.docx]

**Additional File 1: Tables S1-S3**

# Table S1

*German translation of the Long Covid Stigma Scale.*

| **Enacted Stigma Items** | |  | |
| --- | --- | --- | --- |
| 1 | Because of my illness, some people seemed uncomfortable with me | | Wegen meiner Erkrankung schienen sich Menschen in meiner Gegenwart unwohl zu fühlen. |
| 2 | Because of my illness, some people were unkind to me | | Wegen meiner Erkrankung waren Menschen unfreundlich zu mir. |
| 3 | People I care about stopped contacting me after learning I have Long Covid | | Menschen, die mir wichtig sind, haben aufgehört, sich bei mir zu melden, nachdem Sie erfahren haben, dass ich Long COVID habe. |
| 4 | People have acted as if I am dishonest since I have had Long Covid | | Seit ich Long COVID habe, tun die Leute so, als ob ich unehrlich wäre. |
| 5 | I have been treated with less respect than other people are because of Long Covid | | Ich wurde aufgrund der Long COVID-Erkrankung mit weniger Respekt behandelt als andere Menschen. |
| **Internalized Stigma Items** | |  | |
| 6 | I have felt embarrassed about my illness | | Ich habe mich wegen meiner Krankheit geschämt. |
| 7 | I have felt embarrassed because of my physical limitations | | Meine körperlichen Einschränkungen waren mir peinlich. |
| 8 | I feel that I have been tainted by Long Covid and am of less value than others because of it | | Ich habe das Gefühl, dass ich wegen Long COVID einen Makel habe und deswegen weniger wert bin als andere. |
| 9 | I have felt like I am very different from other people on account of Long Covid | | Ich habe das Gefühl, dass ich aufgrund meiner Long COVID Erkrankung sehr anders bin als andere Menschen. |
| **Anticipated Stigma Items** | |  | |
| 10 | Many people tend to think Long Covid isn’t a real illness | | Viele Menschen denken tendenziell, dass Long COVID keine echte Krankheit ist. |
| 11 | I feel that some people assume that having Long Covid is a sign of personal weakness | | Ich habe das Gefühl, dass manche Menschen annehmen, dass Long COVID ein Zeichen persönlicher Schwäche ist. |
| 12 | I worry that people with Long Covid lose their jobs when their employers find out | | Ich befürchte, dass Menschen mit Long COVID ihre Arbeit verlieren, wenn der Arbeitgeber davon erfährt. |
| 13 | I worry that people may judge me negatively when they learn I have Long Covid | | Ich bin besorgt, dass Menschen mich negativ beurteilen, wenn sie erfahren, dass ich Long COVID habe. |

*Instruction Item 1-9*: Bei den folgenden Aussagen geht es um Erfahrungen, die Sie im Rahmen Ihrer Long Covid Erkrankung möglicherweise gemacht haben. Bitte geben Sie an wie häufig Sie die folgenden Dinge erlebt haben. *Response categories:* niemals; selten; manchmal; oft; sehr oft

*Instruction Item 10-13:* Bitte geben Sie an in welchem Ausmaß Sie den folgenden Aussagen zustimmen. *Response categories:* stimme gar nicht zu; stimme eher nicht zu; teils, teils; stimme eher zu; stimme völlig zu.

# Table S2

*Item characteristics of the initial six items of perceived psychologization.*

| Selected items | | Mean | Median | SD | IQR | γ_m_ | ω | *P*_m_ | *r*_it_ |
| --- | --- | --- | --- | --- | --- | --- | --- | --- | --- |
| 1 | I get the impression that many people see Long Covid/Post Covid-19 Vaccination Syndrome as a purely mental illness. | 3.17 | 3 | 1.00 | 3-4 | -1.14 | 0.73 | .79 | .68 |
| 2 | I think people who don't have LC/PCVS are unsure whether they can really believe my symptoms. | 2.99 | 3 | 1.05 | 2-4 | -0.92 | 0.21 | .75 | .69 |
| 3 | I don't feel taken seriously when I tell others about my LC/PCVS symptoms. | 2.70 | 3 | 1.12 | 2-4 | -0.59 | -0.39 | .68 | .69 |
| 4 | I feel labeled as mentally ill when I talk about my LC/PCVS symptoms. | 2.84 | 3 | 1.16 | 2-4 | -0.75 | -0.35 | .71 | .75 |
| Excluded items | |  |  |  |  |  |  |  |  |
|  | I feel helpless when my LC/PCVS symptoms are attributed to psychological causes. | 3.37 | 4 | 1.03 | 3-4 | -1.75 | 2.35 | .84 | .55 |
|  | It annoys me when my LC/PCVS symptoms are attributed to the psyche. | 3.70 | 4 | 0.70 | 4-4 | -2.86 | 8.92 | .93 | .48 |

Descriptive statistics of six newly designed items about perceived psychologization of the Long Covid Syndrome (LC = 1398) and Post Covid-19 Vaccination Syndrome (PCVS; *N* = 655). IQR interquartile range, SD standard deviation, γ_m_ skewness, ω kurtosis, *P*_m_ item difficulty, *r*_it_ corrected item-total correlations.

# Table S3

*Analysis of sensitivity to semantic confounding between exposure perceived psychologization and mediator stigmatization.*

| **Outcome** | **Exposure- Mediator**  **(*a*)** | **Mediator-Outcome**  **(*b*)** | | **Direct effect**  **(*c*’)** | | **Mediation (*a* * *b*)** | | **Total**  **(*c*’ + *a* * *b*)** | | ***R*²** |  |
| --- | --- | --- | --- | --- | --- | --- | --- | --- | --- | --- | --- |
| Disclosure concerns | .62*** | .34 | *** | .27 | *** | .21 | *** | .48 | *** | .30 | |
| Loss of trust in medicine |  | .18 | *** | .16 | *** | .11 | *** | .27 | *** | .14 | |
| Life satisfaction |  | -.24 | *** | .03 |  | - .15 | *** | - .12 | *** | .08 | |

Results of the mediation analysis under exclusion of Long Covid Stigma Scale items 10 and 11. We report standardized regression coefficients with asterisks representing *p*-values corrected for multiple testing (three tests; Bonferroni’s procedure).

*** *p* < .001.
