# Supplementary material for: “Have you considered that it could be burnout?”—psychologization and stigmatization of self-reported long COVID or post-COVID-19 vaccination syndrome
Source: BMC Med. 2025 Aug 20;23:488. doi: 10.1186/s12916-025-04335-0 (PMC12366089; doi:10.1186/s12916-025-04335-0)
Supplement: Supplementary file 2 — Additional File 2: Additional information on Methods. [file 12916_2025_4335_MOESM2_ESM.docx]

**Additional File 2: Additional information on Methods**

**1. Additional information *survey instruments*:**

*Adaption of the scale format LCSS:* We adapted the anchors of the 5-point response scale for some of the items to ensure a close fit between item content and response scale. For example, for the item “I worry that people with Long Covid lose their jobs when their employers find out”, we changed the original frequency scale (ranging from “never” to “always”) to an extent of agreement scale (ranging from “strongly disagree” to “strongly agree”).

**2. Additional information *analysis sample*:**

One participant in the LC group stated their initial date of Covid-19 infection as being less than four weeks ago. Since four weeks is the minimal time to consider Long Covid, this participant was also excluded. Within the PCVS sample, 11 participants indicated that they received their vaccination before December 2020, and were excluded due to December 2020 being the time when the EU approved the first Covid-19 vaccinations.

We excluded three datasets where the participants did not pass an attention check incorporated in the middle of the survey (respondents were presented with five different animals and asked to check the option “giraffe”).

**3. Additional information *statistical analysis***

*H3:* As primary operationalization of the exposure we chose perceived psychologization (four-item mean score), which reasonably will exhibit a higher reliability than our single item on experienced psychologization. For the mediator, we binned the LCSS mean score into eight equidistant categories to ensure estimation model stability. We modelled all three outcomes simultaneously to allow for an assessment of their interrelationships (Rijnhardt, 2021). Disclosure concerns entered as two-item mean score, loss of trust as single item rating, and the rating of life satisfaction was binned to five equidistant categories to ensure estimation model stability. In deference to model efficiency, we included only covariates, which in bivariate tests demonstrated a potential to confound the relationships between exposure, mediator, and/or outcomes in the full sample or in the diagnostic subgroups (LC and PCVS). Consequently, we controlled all model paths for age (continuous), gender (non-binary excluded), education (categorical: < 10 years, 10 years, > 10 years), history of mental illness (binary, “don’t know” and ”don´t want to say” considered as “yes”), severity of LC or PCVS symptoms during the worst phase (sum score), and whether the diagnosis of the syndrome had been established by a medical doctor (binary).

All model constituents were modeled as manifest variables under the assumption of error free measurement (SEM subtype path analysis). To account for non-normality of the mediator and the outcomes, we used a diagonally weighted least squares estimator and robust standard errors based on the full weight matrix. We tested hypothesis H3 using the conservative Sobel test (MacKinnon, 2002; Sobel, 1982) for the mediation term, which we defined as the effect of the exposure on the mediator times the effect of the mediator on the outcome (product of coefficients method).

We checked the robustness of the mediation model established in the full sample by comparing it against several alternate variants. First, sensitivity to the type of the syndrome (LC and PCVS) was tested by way of subgroup analyses. Second, robustness of the mediation estimates to semantic confounding between the exposure perceived psychologization and the mediator stigmatization was checked by excluding LCSS items 10 and 11 in the full sample analysis. Both items had shown high loadings (>.7) on the perceived psychologization factor in an exploratory factor analysis with Varimax rotation. Finally, to explore differences between perceived and experienced psychologization, we repeated the mediation analyses in the full sample and diagnosis groups using experienced psychologization as exposure instead. Cases with missing values were deleted listwise (1·5% loss).
