# Supplementary material for: “Have you considered that it could be burnout?”—psychologization and stigmatization of self-reported long COVID or post-COVID-19 vaccination syndrome
Source: BMC Med. 2025 Aug 20;23:488. doi: 10.1186/s12916-025-04335-0 (PMC12366089; doi:10.1186/s12916-025-04335-0)
Supplement: Supplementary file 3 — Additional File 3: Original survey. [file 12916_2025_4335_MOESM3_ESM.docx]

**Additional File 3: Original survey.**

*Note: Original German Questionnaire. English subheadings and notes are marked in yellow.*

1. Study information for participants

Liebe Teilnehmende,

die vorliegende Studie der Universität Leipzig richtet sich an Personen ab 18 Jahren, die von Nachwirkungen einer Covid-19-Infektion (Long Covid) oder einer Impfung gegen Covid-19 (Post-Vac) betroffen sind oder waren. Wir möchten dabei herausfinden, welche Erfahrungen Menschen, die von Long Covid- oder Post-Vac-Symptomen betroffen sind, im Umgang mit Ihrer Erkrankung gemacht haben.

Diese Befragung findet komplett anonym statt und wir sind Ihnen sehr dankbar, wenn Sie so ehrlich wie möglich antworten. Mit Ihrer Teilnahme an der Befragung leisten Sie einen unmittelbaren Beitrag zu aktueller Forschung im Bereich der Wahrnehmung von Menschen mit anhaltenden Symptomen nach einer Covid-Infektion oder -Impfung.

Das Ausfüllen der Befragung nimmt etwa 15-20 Minuten in Anspruch. Ausführliche Informationen zum Ablauf und zum Hintergrund der Studie sowie zur Verarbeitung Ihrer Daten finden Sie in den Studieninformationen, die Sie auf der nächsten Seite herunterladen können. Vielen Dank für Ihre Teilnahme!

1. Filter question: LC or PCVS

| Sind Ihre Symptome nach einer Covid-19 Erkrankung oder nach einer Impfung gegen Covid-19 aufgetreten? | O nach Covid-Erkrankung  O nach Impfung  O keine der beiden Optionen trifft auf mich zu |
| --- | --- |

*Note: Below the questionnaire for LC is presented. The questionnaire for PCVS was identical, with only “Long Covid” being replaced by “Post Vac.”*

1. Age and Gender

| Wie alt sind Sie? | Alter in Jahren |
| --- | --- |
| Bitte geben Sie Ihr Geschlecht an | O männlich  O weiblich  O anderes, und zwar: ______ (offenes Textfeld) |

1. Stigmatization

Long Covid Stigma Scale (Pantelic et al., 2022), German version (please see *Additional File 1*, *Table S1* for details on our German translation and the English original version)

| Bei den folgenden Aussagen geht es um Erfahrungen, die Sie im Rahmen Ihrer Long Covid Erkrankung möglicherweise gemacht haben. Bitte geben Sie an wie häufig Sie die folgenden Dinge erlebt haben. | 1  niemals | 2  selten | 3  manchmal | 4  oft | 5  sehr oft |
| --- | --- | --- | --- | --- | --- |
| Wegen meiner Erkrankung schienen sich Menschen in meiner Gegenwart unwohl zu fühlen. |  |  |  |  |  |
| Wegen meiner Erkrankung waren Menschen unfreundlich zu mir. |  |  |  |  |  |
| Menschen, die mir wichtig sind, haben aufgehört, sich bei mir zu melden, nachdem Sie erfahren haben, dass ich Long Covid habe. |  |  |  |  |  |
| Seit ich Long Covid habe, tun die Leute so, als ob ich unehrlich wäre. |  |  |  |  |  |
| Ich wurde aufgrund der Long Covid-Erkrankung mit weniger Respekt behandelt als andere Menschen. |  |  |  |  |  |
| Ich habe mich wegen meiner Krankheit geschämt. |  |  |  |  |  |
| Meine körperlichen Einschränkungen waren mir peinlich. |  |  |  |  |  |
| Ich habe das Gefühl, dass ich wegen Long Covid einen Makel habe und deswegen weniger wert bin als andere. |  |  |  |  |  |
| Ich habe das Gefühl, dass ich aufgrund meiner Long Covid-Erkrankung sehr anders bin als andere Menschen. |  |  |  |  |  |
| Bitte geben Sie an in welchem Ausmaß Sie den folgenden Aussagen zustimmen. | 1  stimme gar nicht zu | 2  stimme eher nicht zu | 3  teils, teils | 4 stimme eher zu | 5 stimme völlig zu |
| Viele Menschen denken tendenziell, dass Long Covid keine echte Krankheit ist. |  |  |  |  |  |
| Ich habe das Gefühl, dass manche Menschen annehmen, dass Long Covid ein Zeichen persönlicher Schwäche ist. |  |  |  |  |  |
| Ich befürchte, dass Menschen mit Long Covid ihre Arbeit verlieren, wenn der Arbeitgeber davon erfährt. |  |  |  |  |  |
| Ich bin besorgt, dass Menschen mich negativ beurteilen, wenn sie erfahren, dass ich Long Covid habe. |  |  |  |  |  |

1. Perceived Psychologization

| Bitte geben Sie an in welchem Ausmaß Sie den folgenden Aussagen zustimmen. | 1  stimme gar nicht zu | 2  stimme eher nicht zu | 3  teils, teils | 4 stimme eher zu | 5 stimme völlig zu |
| --- | --- | --- | --- | --- | --- |
| Ich habe den Eindruck, dass viele Menschen Long Covid als rein psychische Erkrankung ansehen. |  |  |  |  |  |
| Ich fühle mich hilflos, wenn meine Long Covid-Symptome auf psychische Ursachen zurückgeführt werden. |  |  |  |  |  |
| Ich glaube, Menschen, die kein Long Covid haben, sind unsicher, ob sie mir meine Symptome wirklich glauben können. |  |  |  |  |  |
| Ich fühle mich nicht ernst genommen, wenn ich anderen über meine Long Covid-Symptome berichte. |  |  |  |  |  |
| Es ärgert mich, wenn meine Long Covid-Beschwerden mit der Psyche begründet werden. |  |  |  |  |  |
| Ich fühle mich als psychisch krank abgestempelt, wenn ich von meinen Long Covid-Symptomen erzähle. |  |  |  |  |  |

1. Information on acute infection

| Wann war die Covid-19-Infektion, in deren Folge Sie die Long Covid-Symptome entwickelt haben?  [Wenn Sie sich nicht genau erinnern können, schätzen Sie bitte den Zeitpunkt. Bitte geben Sie das Jahr an:] | O 2020  O 2021  O 2022  O 2023  O 2024 |
| --- | --- |
| Bitte geben Sie den Monat an: | Monat |
| Wie wurde die Covid-19-Infektion festgestellt?  [Mehrfachantwort möglich] | O SARS-CoV-2-PCR-Test  O Antigen-Schnelltest  O aus Symptomen geschlussfolgert  O anderes (wenn ja, welche: ______________) |
| Wie war der Verlauf dieser akuten Covid-19-infektion, in deren Folge Ihre Long Covid-Symptome aufgetreten sind? | O asymptomatisch (keinerlei Symptome, aber positiver Test)  O leicht (nur leichte Symptome)  O moderat (ausgeprägte Symptome)  O schwer (schwere Symptome, möglicherweise sogar Krankenhausaufenthalt inkl. Lungenentzündung/Sauerstoffgabe, aber ohne Beatmung)  O kritisch (Krankenhausaufenthalt inkl. Intensivstation mit Beatmung) |

1. Information on Long Covid

| Wann sind Ihre Long Covid-Symptome erstmalig aufgetreten?  [Wenn Sie sich nicht genau erinnern können, schätzen sie bitte den Zeitpunkt. Bitte geben Sie das Jahr an:] | #2020 #2021 #2022 #2023 #2024 |
| --- | --- |
| Bitte geben Sie den Monat an: | #Januar #Februar #März #April #Mai #Juni #Juli #August #September #Oktober #November #Dezember |
| Von welchen Symptomen waren Sie in der schlimmsten Phase Ihrer Long Covid-Erkrankung betroffen?  [Mehrfachantwort möglich] | # Geschmacks- oder Geruchsstörungen  # Fatigue (chronische Erschöpfung, Müdigkeit)  #Mangelnde körperliche Belastbarkeit (z.B. Kurzatmigkeit bei Belastung, volle Leistungsfähigkeit nicht wieder erreicht)  # Verschlechterung der Beschwerden nach Alltagsaktivitäten (sog. Post exertionelle Malaise)  #Kopfschmerzen  # Gelenk- oder Muskelschmerzen  # Beschwerden im Hals-, Nasen- oder Ohrenbereich (z.B. Heiserkeit, Schmerzen oder Kratzen im Hals, laufende Nase)  # Lungen- oder Atembeschwerden (z.B. Husten, pfeifende oder keuchende Atmung)  # Herzbeschwerden (z.B. Extrasystolen, Herzrasen, Brustschmerzen)  # Schwindel beim Aufstehen, längeren Stehen  # Magen-Darmbeschwerden (z.B. Bauchschmerzen, Durchfall, Erbrechen, Übelkeit)  # Beschwerden oder Auffälligkeiten des Nervensystems oder Gedächtnis (z.B. Wortfindungsstörung, Konzentrationsstörung)  # Hautbeschwerden (z.B. Haarausfall, Ausschlag, Juckreiz)  # Infektionszeichen (z.B. Schüttelfrost, Fieber, grippeartiges Gefühl)  # Schlafstörungen (z.B. Einschlaf- oder Durchschlafstörungen, Schlaf nicht erholsam)  #andere, und zwar: |
| Von welchen anderen Symptomen? | Text |
| Von welchem Symptomen sind Sie **aktuell noch** betroffen?  [Mehrfachantwort möglich] | # Geschmacks- oder Geruchsstörungen  # Fatigue (chronische Erschöpfung, Müdigkeit)  #Mangelnde körperliche Belastbarkeit (z.B. Kurzatmigkeit bei Belastung, volle Leistungsfähigkeit nicht wieder erreicht)  # Verschlechterung der Beschwerden nach Alltagsaktivitäten (sog. Post exertionelle Malaise)  #Kopfschmerzen  # Gelenk- oder Muskelschmerzen  # Beschwerden im Hals-, Nasen- oder Ohrenbereich (z.B. Heiserkeit, Schmerzen oder Kratzen im Hals, laufende Nase)  # Lungen- oder Atembeschwerden (z.B. Husten, pfeifende oder keuchende Atmung)  # Herzbeschwerden (z.B. Extrasystolen, Herzrasen, Brustschmerzen)  # Schwindel beim Aufstehen, längeren Stehen  # Magen-Darmbeschwerden (z.B. Bauchschmerzen, Durchfall, Erbrechen, Übelkeit)  # Beschwerden oder Auffälligkeiten des Nervensystems oder Gedächtnis (z.B. Wortfindungsstörung, Konzentrationsstörung)  # Hautbeschwerden (z.B. Haarausfall, Ausschlag, Juckreiz)  # Infektionszeichen (z.B. Schüttelfrost, Fieber, grippeartiges Gefühl)  # Schlafstörungen (z.B. Einschlaf- oder Durchschlafstörungen, Schlaf nicht erholsam)  #andere, und zwar: |
| Von welchen anderen Symptomen? | Text |
| Haben Sie von einem Arzt/einer Ärztin die Diagnose Long bzw. Post Covid gestellt bekommen? | #ja  #nein  #weiß nicht |

1. Disclosure concerns

| Ich bin vorsichtig damit, wem ich von meiner Long Covid-Erkrankung erzähle. | O 1 (stimme überhaupt nicht zu)  O 2  O 3  O 4  O 5 (stimme voll und ganz zu) |
| --- | --- |
| Ich bereue, dass ich bestimmten Leuten von meiner Long Covid-Erkrankung berichtet habe. | O 1 (stimme überhaupt nicht zu)  O 2  O 3  O 4  O 5 (stimme voll und ganz zu) |

1. Experienced Psychologization

| Haben Sie es schon mal erlebt, dass Ihre Long Covid-Symptome vor allem einer psychischen Erkrankung zugeschrieben wurden (z.B. Burnout, Depression)? | O 1 (noch nie)  O 2 (selten)  O 3 (manchmal)  O 4 (oft)  O 5 (sehr oft) |
| --- | --- |
| [Wenn 2 oder höher:] Bei wem haben Sie das erlebt?  [Mehrfachantwort möglich] | O Familienangehörige  O enge Freund:innen/gute Bekannte  O Kolleginnen & Kollegen  O Ärztinnen/Ärzte und andere medizinisch/therapeutisch Tätige  O Behörden  O andere (wenn ja, welche: _______________) |
| [Verbundenes Item dazu, für jeden angekreuzten Bereich:] Wie sehr hat Sie das emotional belastet? | O 1 (gar nicht)  O 2  O 3  O 4  O 5 (sehr stark) |

1. Loss of trust

| Hat sich Ihr Vertrauen in die Medizin durch Ihre Erfahrungen während Ihrer Long Covid Erkrankung verändert? | Schieberegler, 9-stufige Skala,  von „stark abgenommen“ -> Mittelmarkierung: „gleich geblieben“ -> bis „stark zugenommen“ |
| --- | --- |

1. Attention Check

| Bitte kreuzen Sie das Wort „Giraffe“ an. | O Nilpferd  O Katze  O Taube  O Giraffe  O Hamster |
| --- | --- |

*Note: For instruments by other authors, we refer to the original sources only in order to honor orignal authorship.*

1. Screening for symptoms of depression and anxiety

PHQ-4 (Kroenke et al., 2009)

1. Life satisfaction

L1 (Beilerlein et al., 2014)

1. Self-esteem

Brief Rosenberg Self-Esteem Scale (Monteiro et al., 2022)

1. Loneliness

Three-Item Loneliness Scale (Hughes et al., 2004)

1. Previous mental illness

| Waren Sie schon einmal wegen einer psychischen Krankheit in Behandlung? | O Ja  O Nein  O Möchte ich nicht sagen  O Weiß nicht |
| --- | --- |

1. Sociodemographics

| In welchem Bundesland leben Sie aktuell? | O Ich lebe nicht in Deutschland  O Baden-Württemberg  O Bayern  O Berlin  O Brandenburg  O Bremen  O Hamburg  O Hessen  O Mecklenburg-Vorpommern  O Niedersachsen  O Rheinland-Pfalz  O Nordrhein-Westfalen  O Saarland  O Sachsen  O Sachsen-Anhalt  O Schleswig-Holstein  O Thüringen |
| --- | --- |
| Sie haben angegeben, dass Sie aktuell nicht in Deutschland leben. In welchem Land leben Sie aktuell? | Textfeld |
| Welchen höchsten allgemein bildenden Schulabschluss haben Sie? | O noch Schüler:in ohne Abschluss  O von der Schule abgegangen ohne Hauptschulabschluss (ohne Volksschulabschluss)  O Hauptschulabschluss (Volksschulabschluss)  O Realschulabschluss (Mittlere Reife)  O Abschluss der Polytechnischen Oberschule 10. Klasse (vor 1965: 8. Klasse)  O Fachhochschulreife, Abschluss Fachoberschule  O allgemeine/fachgebundene Hochschulreife/ Abitur (Gymnasium/EOS, auch EOS mit Lehre)  O weiß nicht  O will ich nicht angeben  O einen anderen Schulabschluss (welchen? _____) |
| Falls Sie momentan noch Schüler:in ohne Abschluss sind, welchen Abschluss streben Sie an? | O Hauptschulabschluss (Volksschulabschluss)  O Realschulabschluss, Mittlere Reife, Fachschulreife  O Fachhochschulreife, Abschluss Fachoberschule  O Abitur, allgemeine oder fachgebundene Hochschulreife  O weiß nicht  O will ich nicht angeben  O einen anderen Schulabschluss (welchen? _____) |
| Sind Sie zur Zeit erwerbstätig? | O ja, ich bin vollzeiterwerbstätig mit 35 Stunden und mehr pro Woche  O ja, ich bin teilzeiterwerbstätig mit 15 bis 34 Stunden pro Woche  O ja, ich bin teilzeit-/stundenweise erwerbstätig mit unter 15 Stunden pro Woche  O nein, ich bin nicht erwerbstätig  O weiß nicht  O will ich nicht angeben |
| In welcher beruflichen Stellung sind Sie derzeit beschäftigt bzw. waren Sie hauptsächlich beschäftigt? | O Angestellte:r  O Arbeiter:in (auch in der Landwirtschaft)  O Beamte:r (einschließlich Richter:in und Berufssoldat:in)  O Selbstständige:r (einschließlich mithelfende Familienangehörige)  O Student:in, Auszubildende:r Praktikant:in  O keines davon trifft auch mich zu |
| Angestellte:r, und zwar… | O mit ausführender Tätigkeit nach allgemeinen Anweisungen  O mit einer qualifizierten Tätigkeit, die ich nach Anweisung erledige  O mit eigenständiger Leistung in verantwortlicher Tätigkeit bzw. mit Fachverantwortung für Personal  O Abteilungsleiter:in bzw. Meister:in im Angestelltenverhältnis  O mit umfassenden Führungsaufgaben und Entscheidungsbefugnissen |
| Arbeiter:in, und zwar… | O ungelernte Arbeiter:in  O angelernte Arbeiter:in  O Facharbeiter:in  O Vorarbeiter:in, Kolonnenführer:in  O Meister:in, Polier:in, Brigardier:in |
| Beamte:r, und zwar… | O im einfachen Dienstag  O im mittleren Dienst  O im gehobenen Dienst  O im höheren Dienst, Richter:in |
| Selbstständig, und zwar… | O keine weiteren Mitarbeiter:innen  O 1 bis 4 Mitarbeiter:innen  O 5 und mehr Mitarbeiter:innen |
| Ausbildung/Studium, und zwar… | O als kaufmännisch-technische:r Auszubildende:r  O als gewerbliche:r Auszubildende:r  O in sonstiger Ausbildungseinrichtung  O Studium |
| Wenn Sie momentan nicht erwerbstätig sind, welcher Gruppe gehören Sie an? | O in Schule, Ausbildung oder Studium  O arbeitslos (Empfänger:in von Arbeitslosengeld I)  O arbeitslos (Empfänger:in von Arbeitslosengeld II, Bürgergeld)  O arbeitslos (ohne Empfang von Bezügen)  O Hausfrau, Hausmann  O Wehr-/ Zivildienstleistende:r oder im freiwilligen sozialen Jahr  O in Mutterschutz oder Erziehungsurlaub  O im Vorruhestand, Rente oder Pension  O keins davon trifft auf mich zu  O weiß nicht  O will ich nicht angeben |
| Wie viele Personen leben ständig in Ihrem Haushalt? [Hinweis: Zu einem Haushalt zählen alle Personen, die dort gemeinsam wohnen und wirtschaften. Denken Sie dabei bitte auch an alle ständig, das heißt mit hauptsächlichem Wohnsitz, in Ihrem Haushalt lebenden Kinder.] | O 1  O 2  O 3  O 4  O 5  O 6  O 7  O 8  O 9  O 10  O mehr als 10 |
| Falls mindestens zwei Personen (Sie selbst eingeschlossen) in Ihrem Haushalt leben: Wie viele Personen in Ihrem Haushalt sind jünger als 14 Jahre? | O Keine Person unter 14 Jahren im Haushalt  O 1  O 2  O 3  O 4  O 5  O 6  O 7  O 8  O 9  O 10  O mehr als 10 Personen |
| Wie hoch ist aktuell das monatliche Nettoeinkommen Ihres Haushalts? | O kein monatliches Einkommen  O unter 150 Euro  O 150 bis unter 400 Euro  O 400 bis unter 500 Euro  O von 500€ 🡪 4.000€ in 250er Schritten  O von 4.000€ 🡪 7.500€ in 500er Schritten  O 7.500 bis unter 10.000 Euro  O 10.000 bis unter 20.000 Euro  O 20.000 Euro und mehr  O will ich nicht angeben |

1. End of the questionnaire: open text box for additional comments

| Falls Sie abschließend noch Anmerkungen, Ergänzungen o.ä. haben, können Sie uns an dieser Stelle gerne einen Kommentar hinterlassen. Vielen Dank! | Textfeld |
| --- | --- |
